# Development and Characterization of Multi-Alkali Antimonide Photocathodes for High-Brightness RF Photoinjectors

**DOI:** 10.3390/mi14061182

**Published:** 2023-05-31

**Authors:** Sandeep Kumar Mohanty, Mikhail Krasilnikov, Anne Oppelt, Frank Stephan, Daniele Sertore, Laura Monaco, Carlo Pagani, Wolfgang Hillert

**Affiliations:** 1 Deutsches Elektronen-Synchrotron DESY, 15738 Zeuthen, Germany; 2 Istituto Nazionale di Fisica Nucleare—LASA, 20090 Segrate, Italy; 3 Institute of Experimental Physics, University of Hamburg, 22607 Hamburg, Germany; 4 Dipartimento di Fisica, Università degli Studi di Milano, 20122 Milano, Italy

**Keywords:** multi-alkali photocathodes, quantum efficiency, optical properties, density function theory

## Abstract

Due to their excellent photoemissive properties, especially low thermal emittance and high sensitivity in the green wavelength, multi-alkali antimonide photocathodes, in particular, cesium–potassium–antimonide, emerged as prominent photoemissive materials for the electron sources of high-repetition-rate FEL applications. To explore its feasibility of operating in a high-gradient RF gun, DESY collaborated with INFN LASA to develop multi-alkali photocathode materials. In this report, we describe the recipe of K-Cs-Sb photocathodes, which were grown on a Mo substrate by varying the foundational Sb layer thickness using sequential deposition techniques. This report also illustrates the information regarding the film thickness, substrate temperature, deposition rate, and its possible effects on the photocathode’s properties. In addition, the influence of temperature on the cathode degradation is also summarized. Furthermore, in the framework of density functional theory (DFT), we investigated the electronic and optical properties of the K_2_CsSb material. The optical properties, such as dielectric function, reflectivity, refracting index, and extinction coefficient, were evaluated. The correlation between the calculated and measured optical properties, such as reflectivity, provides a better and more efficient strategy to rationalize and understand the photoemissive material’s properties.

## 1. Introduction

The modern advancement of electron sources made it possible to develop some pioneering applications of electron accelerators, such as X-ray free-electron lasers (FELs) and energy recovery linacs (ERLs), which produce high-brightness, ultra-short pulses of photon radiation with a dedicated pulse structure [1,2,3]. To obtain these features, a high-QE and low-intrinsic-emittance-based photocathode plays a key role. In recent years, semiconductor materials mainly belonging to the alkali antimonides have been widely used for these applications due to their properties, such as high efficiency in the visible range of the spectrum, reasonable lifetime, and a sub-picosecond response time [4,5]. However, in the semiconductor family, cesium telluride (Cs_2_Te) photocathodes are the most popular for accelerator applications due to their robustness in the high-gradient environment. Since these films are sensitive to the UV range with a typical QE of 10% or more, the photocathode laser systems are more demanding, as it is necessary to go to the third or fourth harmonic from the fundamental infrared laser light (IR). The conversion efficiency over several stages is naturally very small, which requires high initial laser powers. Furthermore, this kind of laser system is generally very complex, which increases the operation costs [6]. As a potential solution, gallium arsenide (GaAs) has a credible high QE (~10%) in the visible spectrum, but has a longer response time (tens of ps) and high sensitivities under a radio frequency (RF) environment [7]. By comparison, alkali antimonide photocathodes, in particular, cesium–potassium–antimonide, have a reasonable QE of up to ~10% in the green wavelength range, a prompt response time, and low thermal emittance, making it a promising candidate to generate a high brightness beam for light source applications [8]. Nevertheless, alkali antimonide compounds need a better vacuum quality (i.e., 10^−11^ mbar) range than Cs_2_Te (i.e., 10^−10^ mbar) to limit QE degradation [9]. The utilization of this photocathode has so far been successfully demonstrated in various DC and continuous wave (CW) guns at low gradients (<20 MV/m) [10,11], and parameters such as QE and thermal emittance were found to be very promising. Recently, it was demonstrated that this kind of cathode can sustain a month of long continuous operation inside a QWR SRF gun [5]. However, improving the brightness of the electron beam in next-generation CW guns will require even higher cathode gradients (30–40 MV/m) for various applications. Therefore, our current research domain was mainly focused on developing multi-alkali photocathode materials and exploring their feasibility for high-gradient operation at the PITZ RF gun for a future upgrade of the European XFEL facility.

The Photo Injector Test facility at DESY, Zeuthen site (PITZ), is mainly dedicated to the development and optimization of high-brightness electron sources for free-electron lasers (FELs), such as FLASH and European XFEL [12]. The PITZ photoinjector system is currently being operated with a UV-sensitive Cs_2_Te photocathode. A normal conduction L-band radio frequency (RF) gun cavity generates electron beams with a beam energy up to 7 MeV and bunch charges up to several nC, with a peak acceleration field of ∼60 MV/m. For the development part of the K-Cs-Sb-based photocathodes, DESY collaborated with INFN LASA, which has long-standing experience in studying and growing semiconductor-based photocathode material and Cs_2_Te photocathodes produced at INFN LASA are regularly used in different facilities, such as FLASH, PITZ, and LBNL. 

The alkali antimonide photocathode’s growth process is very challenging due to its sensitivity to temperature, pressure, and deposition rate. Therefore, the reliable growth process of this type of material is primarily based on the recipe obtained by trial and error [13]. In this study, we developed our cathodes with a sequential deposition method on a Mo substrate and optimized the recipe by changing the substrate temperatures, thicknesses, deposition rates, etc. The following section of this report describes an overview of different cathode growth recipes (produced in the R&D system) and the plausible effects of film thickness, substrate temperature, and deposition rate on the photocathode’s properties. 

## 2. Materials and Methods

### 2.1. Photocathode Laboratory

To develop a reproducible recipe of alkali antimonide photocathodes, we generally grow the films in the “R&D” preparation chamber at LASA, where we have implemented our past gathered data related to these materials [9]. Recently, a new dedicated preparation system was built to deposit alkali antimonide films on INFN Mo plugs for their testing in the PITZ RF gun [14,15]. Although this new system is thought to be a “production” unit that supplies cathodes to photoinjectors, we can also use it to support the R&D activity by producing cathodes with different recipes and techniques, such as the co-evaporation of alkali metals. 

The “R&D” preparation system consists of two interconnected chambers, which are used for the cathode growth and storage of samples [16]. The cathode preparation chamber is maintained at a base pressure in the 10^−11^ mbar range, which is provided by eight SAES Getters NEG St707^®^ modules and a 400 L/s ion pump. This system is also connected with a μ-metal shielded chamber, which hosts a time-of-flight (TOF) spectrometer that is used for thermal emittance measurements [17]. During the deposition process, the Mo sample is heated to a specific temperature while compounds are sequentially evaporated by dispensers. In the meantime, the evaporation rate is monitored by a microbalance, and both the real-time photocurrent and reflected power of the film are measured through the sapphire viewport. 

The new UHV preparation system (which is dedicated to alkali photocathodes production) has been equipped with the standard UHV devices (pressure gauges, a residual gas analyzer, and manipulators), two vacuum pumps (a combination of a sputter-ion pump and a Nex-Torr from SAES Getters), and a newly designed Mo plug heater.

A laser drive light source (LDLS) system accompanied by a set of dedicated optical filters (in the range of 239 nm to 436 nm) and a monochromator is used to measure the spectral response of the photocathode. One Ar+ and three He-Ne lasers are used additionally to cover the range from 457 nm to 633 nm. The “production” system is also equipped with a multi-wavelength diagnostic system (in the range from 254–690 nm) to measure the real-time photocurrent and reflectivity at different wavelengths.

### 2.2. Photocathode Preparation

So far, a total number of 8 K-Cs-Sb, 2 Na-K-Sb-Cs, and 2 Na-K-Sb photocathodes have been produced in the “R&D” preparation system. In parallel, 6 K-Cs-Sb photocathodes are deposited in the new “Production” system. In this report, we mainly discuss the preparation and results of multi-alkali photocathodes that were obtained from the “R&D” preparation system. Owing to the better handling, the photoemissive materials were produced on a simplified Mo substrate in the “R&D” preparation chamber. These samples were prepared from a thin slab of high-purity molybdenum (99.95%) through machining. Afterward, the samples were polished to a mirror-like finish (reflectivity > 54% @ 543 nm w.r.t. 57% theoretical [18]) to allow for reflectivity measurements during and after the photocathode growth. Finally, the samples were ultrasonically cleaned before loading them into the UHV system. Before the deposition, each sample was heated up to 450 °C for at least one hour to remove possible residuals on the surface.

A custom-made source for Sb and commercially available dispensers for Cs, Na, and K were used for the deposition. Each source was calibrated to have a particular deposition rate (usually 1 nm/min) during the cathode growth by positioning a microbalance at the same location as the substrate. Once the deposition rate was achieved, the microbalance was moved out of the axis, and the rate was continuously measured to have a cross-calibration that was used during the real evaporation process. The calibration was repeated before each growth process. In order to optimize and better understand the photo emissive film’s properties, we monitored the real-time optical spectra, such as the photocurrent and reflected power during the growth at the green wavelength (i.e., 543 nm). After production, the film was fully characterized by measuring the spectral response and reflectivity in a wider frequency range. The optical spectra of these photocathodes provided a rich source of information on their electronic and film properties. Note that 514 nm was the available wavelength from the PITZ photocathode drive laser, which was the second harmonic of the fundamental laser light. 

## 3. Results

### 3.1. Fabrication Recipes

The basic fabrication process of alkali antimonide photocathodes consisted of a three-step sequential deposition procedure [16,19,20], in which an Sb film was initially deposited on the substrate, then K was sequentially evaporated (until the photocurrent reached the peak) and allowed to react with the Sb film. Finally, by evaporating Cs (until the photocurrent was close to saturation) on the K-Sb film, a K-Cs-Sb compound was formed. To investigate the effect of different thicknesses on the cathode’s properties, we decided to grow two different Sb thickness families (i.e., thin as 5 nm and thick as 10 nm), followed by K and Cs. Moreover, to study the effects of different temperatures and deposition rates on photocathode’s properties, we grew our cathodes with different setup temperatures and deposition rates.

**Figure 1:**
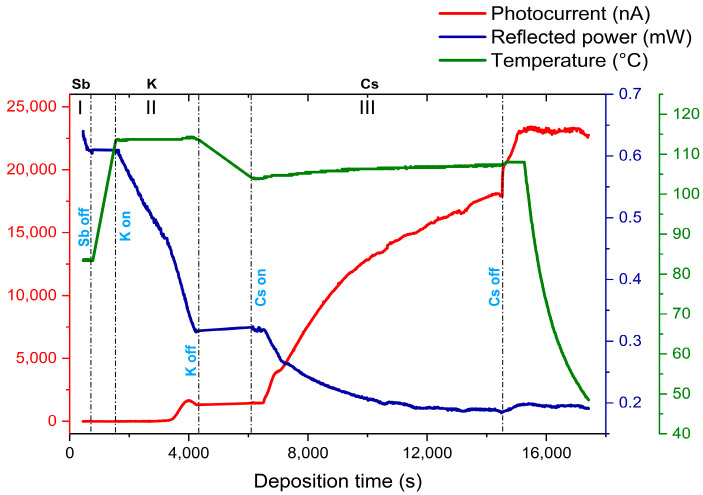
Typical photocurrent and reflected power curve during the deposition. Here, the changes in the reflected power show the behavior of the reflectivity during the growth. In the plot, section I represents the deposition history of Sb, and sections II and III represent the deposition histories of K and Cs, respectively.

During the deposition, a 543 nm He-Ne laser with 1.8 mW of power was constantly illuminated on the cathode’s surface for the real-time photocurrent and reflected power measurement. A bias voltage of 250 V was applied between the anode and the sample to collect the photocurrent from the cathode. A detailed photocurrent (at 543 nm) and reflected power (at 543 nm) history of a typical photocathode (KCsSb-7) during the growth is reported in Figure 1. 

The primary outcome of this experiment was the need for better control over the temperature and its impact on the overall cathode’s properties. The acquaintance of these changes will help us in optimizing the growth recipes to increase reproducibility. The details of the growth procedure at each deposition stage, including the influence of the temperature and thicknesses on the photocathode’s properties of all cathodes, are discussed in a later part of this report. 

As it is reported above, Sb of different thicknesses was initially evaporated on the Mo substrate. During the K deposition process, the evaporated K atoms usually diffuse into the Sb film with subsequent recrystallization into an alkali antimonide structure, generally of M_3_Sb stoichiometry, where M is an alkali metal [21]. Afterward, due to the Cs addition, the alkali antimonide film potentially recrystallizes into the cubic or hexagonal phase of the K_2_CsSb structure, as explained by Schubert et al. [22]. 

We categorized our photocathodes into two typologies: thick (Sb layer was around 10 nm thick) and thin (Sb layer was around 5 nm thick) cathodes depending on the Sb evaporation. The growth parameters of the cathodes produced in the “R&D” system are summarized in Table 1. In our experience with Cs_2_Te [23] and currently with K-Cs-Sb photocathodes, we observed that the ratio of this evaporated thickness (i.e., K/Sb and Cs/Sb in the case of K-Cs-Sb material, see Table 1) was quite reproducible with their definite recipes. Therefore, this information gives a valuable addition to evaluating the film’s properties.

#### 3.1.1. Sb Structure

**Figure 2:**
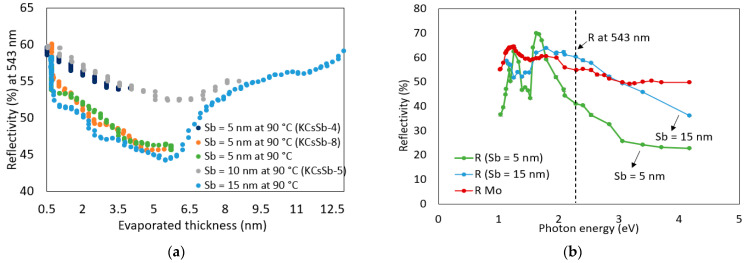
(**a**) Comparison of the real-time reflectivity (at 543 nm) during different Sb depositions. (**b**) Comparison of the full spectral reflectivity between the Sb 5 and Sb 15 nm films on Mo. In (**a**), the real-time reflectivity history during the Sb deposition of some fully grown cathodes (i.e., KCsSb-4, 5, and 8) is presented. The thicknesses in (**a**) represent the evaporated thickness of Sb.

Various studies showed that the evaporation of Sb is an unexpectedly complex process, and its property can influence the cathode performance [13,24]. To explore the effect of different thicknesses on the cathode’s properties, we deposited 5 and 10 nm of Sb on the Mo substrate as a first step to growing the K-Cs-Sb photocathodes. Here, in this section, the results obtained during the Sb deposition of some fully grown cathodes (i.e., KCsSb-4, 5, and 8) are presented. During the deposition of Sb in the “R&D” preparation system, we observed that for both thin (Sb = 5 nm) and thick (Sb = 10 nm) Sb films, the reflectivity (at 543 nm) was decreased. However, for thick Sb films (Sb = 10 nm), after an initial decrease up to about ~6 nm evaporating thickness, the reflectivity again gradually increased. To further investigate this behavior, we grew Sb of two different thicknesses, i.e., 5 and 15 nm, on the Mo substrate. During the process, we monitored the real-time changes in the reflectivity (at 543 nm), as shown in Figure 2a, and measured the full spectral reflectivity (from 254 nm to 1100 nm) at the end of the deposition, as shown in Figure 2b. It can be observed that the reflectivity (at 543 nm) was similarly increased after the film thickness of ~6 nm for the Sb 15 nm film. The reproducibility of this behavior indicates that the film’s properties potentially changed around this particular thickness. In addition, the distinct behavior of the full spectral reflectivity (from 254 nm to 1100 nm) between the Sb 5 and 15 nm films (as shown in Figure 2b) supports this hypothesis. It indicates that Sb was probably in the amorphous form at the initial stage, and later it transformed into a crystalline structure when the film’s thickness increased over ~6 nm. Various authors previously reported the existence of such a transformation by using different surface characterization techniques [21,24,25]. However, the exact value of this transitional thickness may vary, and it depends on various factors, such as the type of substrate, substrate temperature, deposition rate, vacuum quality, and residual gases [24].

#### 3.1.2. K-Sb Structure

**Figure 3:**
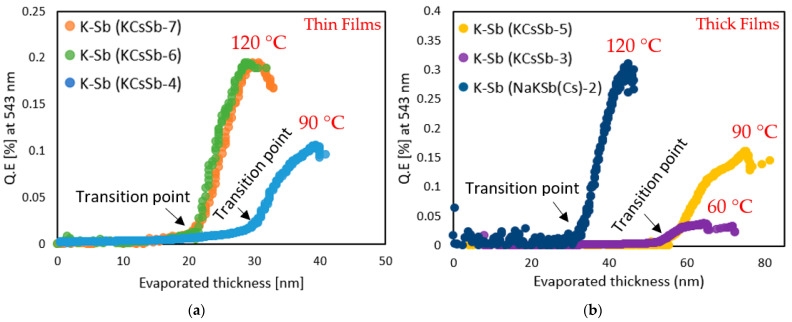

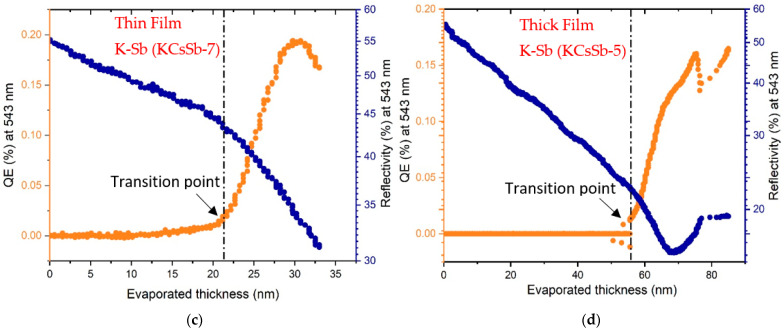
Comparison of the real-time QE evolution (at 543 nm) during the K deposition. (**a**) represents a thin cathode and (**b**) represents a thick cathode. Since the deposition procedure was similar until the K deposition, the K-Sb phase data are included from the NaKSb(Cs)-2 cathode in plot b. The thicknesses presented in the plot represent the evaporated thickness of K. Real-time QE (at 543 nm) and reflectivity (at 543 nm) history during the K deposition: (**c**) thin cathode (i.e., KCsSb-7); (**d**) thick cathode (i.e., KCsSb-5).

The substrate temperature during the K deposition was higher compared with the Cs one because of the lower vapor pressure of K. We typically stopped the K deposition once the photocurrent reached the peak, as shown in Figure 1. We observed that both the substrate temperature and deposition rate played a significant role in terms of the QE for both the thin and thick K-Sb films during the growth. Usually, a relatively higher substrate temperature gives a higher increase in the QE, as shown in Figure 3a,b. Furthermore, we noticed a significant change in the slope of the real-time QE (at 543 nm) and reflectivity (at 543 nm) curves after a certain evaporated thickness (labeled as the transition point), as shown in Figure 3c,d. Indeed, this evolution in the QE after a specific evaporated thickness represented the transition of K-Sb from the amorphous to the crystalline phase [25]. Moreover, we observed that these transition points (amorphous to crystalline) depended on the substrate temperature and usually occurred at a lower amount of evaporated K with increasing substrate temperature, as shown in Figure 3a,b. This effect could unambiguously correlate with a faster diffusion process between Sb and K due to a higher temperature, which induced the crystallization kinetics in the K-Sb film. 

**Figure 4:**
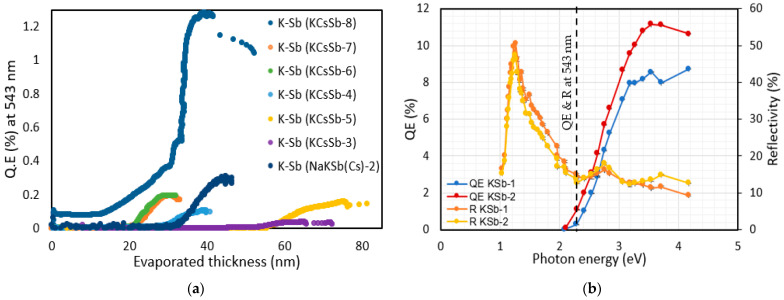
(**a**) Comparison of the real-time QE vs. evaporated thickness (at 543 nm) during the K deposition of all produced K-Sb films in the R&D preparation system. (**b**) Spectral response and reflectivity measurements of the KSb-1 and 2 cathodes.

We also explored the effect of different deposition rates on the K-Sb film’s properties. For the KCSb-8 cathode, we kept the deposition rate at 0.2 to 0.4 nm/min (compared with 1 nm/min before) during the K deposition. Due to the change in the deposition rate and slightly increased temperature (i.e., 130 °C), we observed a significant improvement in the QE of approximately 1.2% at 543 nm for the K-Sb film (compared with the previously measured QEs of approximately 0.2% at 543 nm). A comparison of the real-time QE curve (vs. evaporated thickness) during the K deposition for all produced cathodes in the “R&D” system is presented in Figure 4a. As was previously undertaken for Sb studies, to investigate the reproducibility of the higher QE linked to the lower deposition rate, we grew two K-Sb (KSb-1 and 2) films on the Mo substrate by varying the deposition rate, i.e., the deposition rate of 0.6–1 nm/min for KSb-1 and 0.2–0.4 nm/min for KSb-2 at 130 °C. We observed QEs of approximately 0.3% and 1% for KSb-1 and KSb-2, respectively, at 543 nm. The spectral response and reflectivity of these two K-Sb films are reported in Figure 4b, where it can be observed that the spectral behavior (spectral response and reflectivity) of both cathodes appeared to be similar. From the spectral response curve, the photoemission threshold (energy gap (Eg) + electron affinity (Ea)) was estimated as 2.08 eV in both cases. The similarity in the spectral behavior (reflectivity and spectral response) between the two films indicates that, potentially, a similar compound was formed in both cases. The exact phenomena behind this behavior are not fully understood and require further investigation. A probable explanation is that due to the higher substrate temperature (i.e., 130 °C), the diffusive mobility of the adsorbed molecules became higher in both cathodes, whereas a lower deposition rate decreased the incoming flux of the evaporated molecules (in the case of KSb-2), and it affected the diffusive mobility of the already adsorbed molecules by giving them more time to arrange in a preferred order [26]. This can lead to better crystallinity in the material; hence, a significant improvement in the QE was observed for the KSb-2 cathode. 

**Figure 5:**
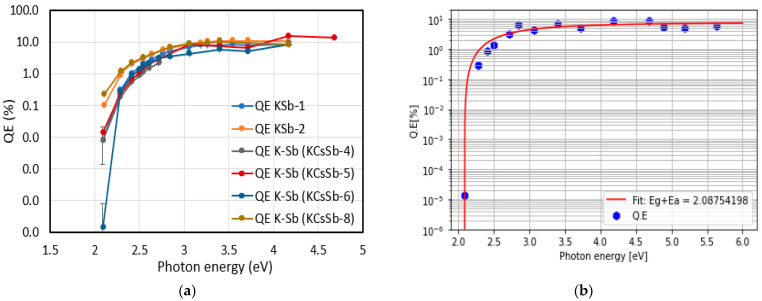
(**a**) Spectral response of all produced K-Sb films in the R&D system. Due to some technical problem, the spectral response of the K-Sb films of cathodes KCsSb-3 and 7 was not measured. (**b**) Spectral response of the K-Sb film (Sb = 5 nm) of cathode KCsSb-6 (blue hexagons). The red curve is the interpolation of the experimental data with Equation (1).

At the end of the K deposition, the film’s color was usually purple, similar to the one reported by Sommer [27]. The spectral response of all produced K-Sb films in the R&D system is presented in Figure 5a. The analysis of the spectral response curve also allowed for estimating the Eg + Ea (energy gap + electron affinity) value of the photocathode, which represents the threshold for photoemission in semiconductor photoemitters. The QE’s dependence near the photon energy threshold can be understood using Equation (1), which was reported by W. E. Spicer [28]:

(1) $$\mathrm{QE}=\frac{G(h\nu )\times {\left[\;h\nu \;-\left(\;Eg+\mathrm{Ea}\right)\right]}^{1.5}}{{\left[\;h\nu \;–\left(\;Eg+\mathrm{Ea}\right)\right]}^{1.5}+\gamma }$$

where hν is the photon energy, Eg + Ea is the sum of the energy gap and electron affinity of the photocathode, and G(hν) is an undefined function of the photon energy. γ can be determined using γ = (α + β)/C, where β and C are constants and α is the absorption coefficient. By fitting Spicer’s model (Equation (1)) into the experimental data (shown in Figure 5b), we estimated that the photoemission threshold value of K-Sb film was 2.08 eV, which is quite similar to the value determined by other works in the literature [24,28].

#### 3.1.3. K-Cs-Sb Structure

**Figure 6:**
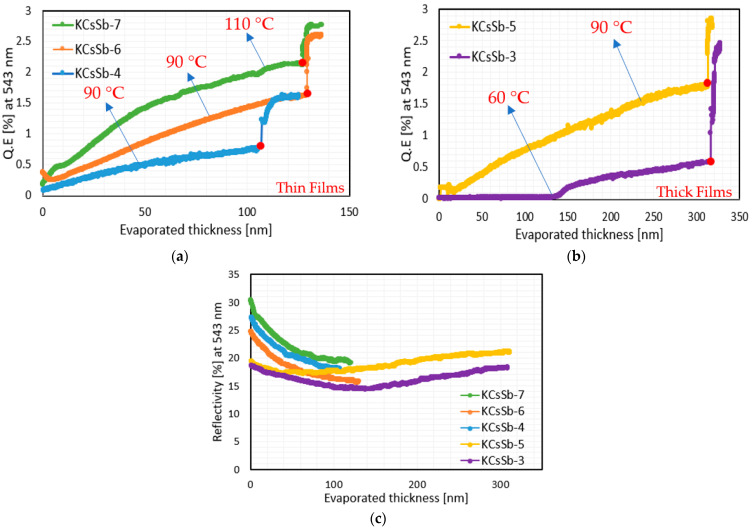
Comparison of the real-time QE (at 543 nm) evolved during Cs deposition. (**a**) represents thin cathodes and (**b**) represents thick cathodes. Due to some technical problem, the Cs deposition was not fully completed for the KCsSb-8 cathode; therefore, it is not included in plot a. Red solid circles in plots a and b show the point where the Cs source was turned off. (**c**) represents the changes in the real-time reflectivity (at 543 nm) during the Cs deposition. The thicknesses presented here in the plot represent the evaporated thickness of Cs.

Finally, a K-Cs-Sb compound was formed by doing the subsequent cesium deposition on the K-Sb films. Cs usually evaporated until the photocurrent is close to the saturation level, as shown in Figure 1. Since the evaporated thickness ratio (i.e., K/Sb and Cs/Sb) was quite reproducible with their definite recipes (shown in Table 1), we also considered the Cs/Sb ratio as an additional criterion to end the Cs deposition. Here, in this phase, we explored the role of substrate temperature and thickness that could impact the cathode’s properties. We observed that the substrate temperature usually played a significant role in dictating the subtle dynamics between the reaction rate of compound formation and the displacement rate of elements already present on the substrate. Usually, the QE depended on the substrate temperature for all six cathodes, as shown in Figure 6a,b. By looking at the Cs/Sb ratio (i.e., evaporated thickness ratio) shown in Table 1, it can be observed that it generally depended upon the initial Sb thickness. Our measurements show that the ratio was higher in thick photocathodes than in thin ones. The real-time evolution of the reflectivity (at 543 nm) of all produced cathodes is reported in Figure 6c. As the Cs evaporation started, we observed that the reflectivity was decreased for both the thin and thick cathodes. However, for thick cathodes, the reflectivity again increased and stabilized close to the end of the evaporation. The difference in such behavior indicated a complex and distinct process of stoichiometric and chemical composition that potentially evolved during the growth of both typologies of cathodes, or it may have been due to the thicknesses of the K-Cs-Sb films, as observed for Cs_2_Te [23]. 

At the end of the Cs evaporation (precisely after turning off the Cs source), we observed a sudden increase in the QE curve for each cathode, as shown in Figure 6a,b. During this event, we did not see any significant changes in the real-time reflectivity. Such a fast QE response certainly could not have been due to the change in the bulk stoichiometry of the compound. This indicates that such an anomaly may have resulted from the subsurface region of the films. In general, due to having a lower melting point (i.e., 28.44 °C) and high vapor pressure [29], a part of the Cs atoms that were deposited on the sample (where the temperature is around 90–110 °C) should have been prone to evaporate. Such a loss of Cs atoms due to evaporation over the substrate temperature of 50 °C was previously reported for K_2_CsSb and Cs_3_Sb photocathodes [30,31]. As a consequence of such evaporation, a temperature-sensitive surface layer may have been formed near the subsurface region of the films during the deposition, which caused a balance between the adsorption (inward cesium being distributed and diffused) and desorption (evaporated cesium from the surface) of cesium atoms near the surface. A sudden decrease in the evaporation rate or any increase in the substrate temperature could upset this balance, and it would have impacted the emission barrier via electron affinity. Table 2 shows the absolute percentage of the QE increase after shutting down the Cs source of all cathodes. It can be observed that the rate of the QE increase could be related to the substrate temperature; for example, in the case of the KCsSb-4, KCsSb-5, and KCsSb-6 cathodes, the percentage of the QE increase was mainly similar and was related to the equal substrate temperature. It is also interesting to note that this increase was primarily correlated to the substrate temperature, independently of the KCsSb (or Sb) evaporated thickness, as shown (in Table 1) for the T = 90 °C case with the thin (KCsSb-4 and KCsSb-6) and thick (KCsSb-5) cathodes; meanwhile, for cathode KCsSb-7, the increase was minimal (compared to other cathodes, i.e., 30%) and could be correlated with the higher substrate temperature. A possible explanation is that due to the higher substrate temperature (for KCsSb-7), the loss of cesium (due to evaporation) might have been compensated by an improved diffusion rate, which probably caused a trade-off between the incoming and outgoing cesium atoms at the surface. By accounting for the above observation, we are improving our cathode recipe and further results will be published in the near future.

**Figure 7:**
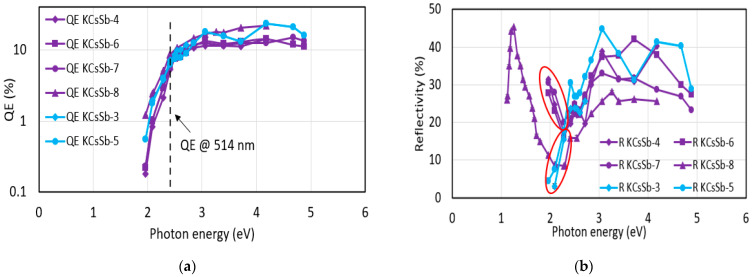
(**a**) Spectral response and (**b**) reflectivity of all the produced K-Cs-Sb cathodes in the R&D system. The spectral response and reflectivity of the thin and thick cathodes are highlighted in violet (with different symbols) and light sky blue (with different symbols), respectively. The difference in the reflectivity at low photon energies is highlighted by the red circles for the thin and thick cathodes. Due to some technical problem, the spectral response measurement was limited from 457 nm to 594 nm for the KCsSb-3 and 4 cathodes.

**Figure 8:**
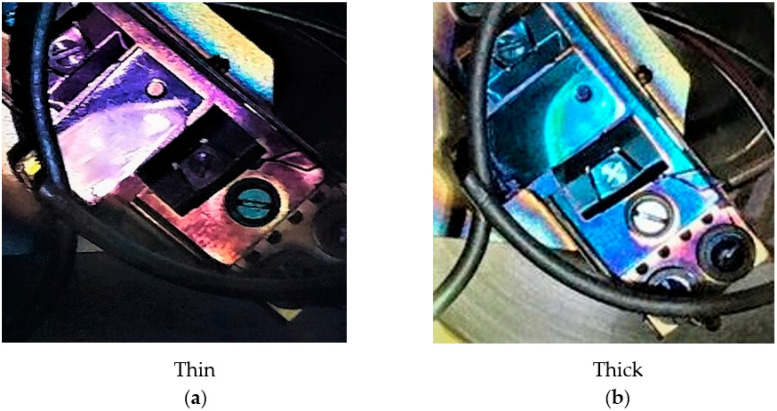
Colors of the photocathodes after the deposition. (**a**) represents a thin cathode and (**b**) represents a thick cathode.

Figure 7 summarizes all the produced cathode’s (in the “R&D” preparation system) spectral responses and reflectivity. The behavior of the spectral response of all the cathodes was comparable. The maximum QE at 514 nm was recorded at ~9% for the KCsSb-8 cathode. However, the comparison of reflectivity shows a difference in the behavior at lower photon energies (from 2.28 eV onward) between the thin and thick cathodes, as shown in Figure 7b. Unfortunately, the reflectivity for a larger range (up to 1100 nm) could only be measured for the KCsSb-8 (thin) cathode. Moreover, we observed two distinct reproducible colors of films, i.e., purple and blue, which were associated with the thin and thick cathodes, respectively, as shown in Figure 8.

**Figure 9:**
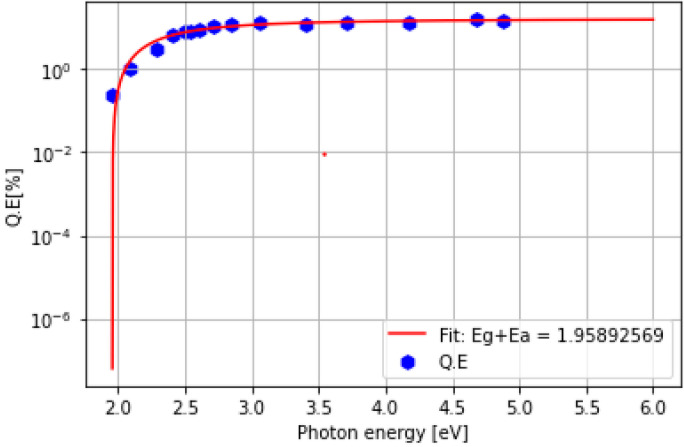
Spectral response of KCsSb-7 (thin) (blue hexagons). The red curve is the interpolation of the experimental data with Equation (1).

To estimate the photoemission threshold value, we fit Equation (1) to our experimental spectral response data (shown in Figure 9) and obtained a value for Eg + Ea that varied between 1.93 and 2 eV from cathode to cathode. The estimated values were quite similar to those determined by other studies in the literature (i.e., Eg = 1.2 eV and Ea = 0.7 eV, Eg + Ea = 1.9 eV) [24]. 

### 3.2. Lifetime Study

All the photocathodes were pretty stable after production. The storage lifetime of these cathodes inside our preparation system (base pressure 10^−11^ mbar) was several months or even years. However, several studies, including our experience, show a short operational lifetime inside an RF gun [14,32]. Several factors, such as chemical poisoning, ion back bombardment, laser heating, and thermal decomposition, could be the potential reasons behind this. To evaluate the effect of laser heating on the cathode properties, we continuously illuminated the cathodes with a He-Ne laser (543 nm wavelength) of 2 mW optical power (power density 0.4 W/cm²) on the cathode’s (KCsSb-7) surface for 15 days (23 µA average emitted current) inside the preparation system. No decay in the QE at 543 nm was observed during this process. Moreover, R. R. Mammei et al.’s simulation studies showed that even at high laser power (~1.5 W), the temperature of a K_2_CsSb photocathode could stay below 50 °C on a Mo substrate [33]. This is due to the better thermal conductivity of the molybdenum material, where the probable heat associated with the absorbed laser power could effectively dissipate from the surface. Therefore, the cathode degradation associated with laser lighting itself is less probable, especially for a cathode on a Mo substrate [32].

**Figure 10:**
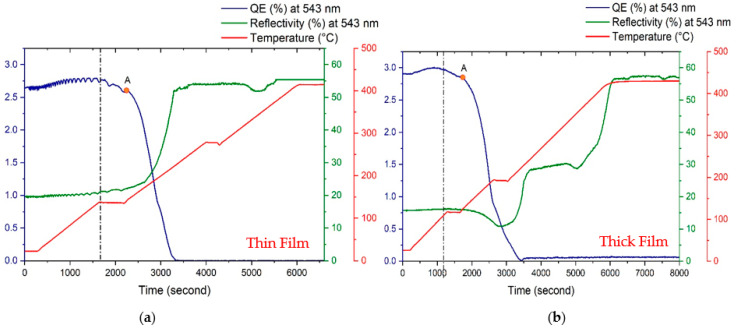
Real-time QE (at 543 nm), reflectivity (at 543 nm), and temperature evolution during the study of the cathode degradation process. (**a**) represents a thin cathode (i.e., KCsSb-6) and (**b**) represents a thick cathode (i.e., KCsSb-5). The dotted line represents the point where the photocurrent started to degrade. “A” denotes the point where the photocurrent started to decrease rapidly.

To understand the effect of the temperature on the cathode’s degradation, we heated our cathodes to 450 °C. Figure 10 shows the evolution of the real-time QE (at 543 nm) and reflectivity (at 543 nm) for thin (KCsSb-6) and thick (KCsSb-5) cathodes during this process. As can be observed, the QE initially increased slightly but later decreased when the substrate temperature went beyond 100 °C for both cathodes. Moreover, a rapid decrease in the QE (at point A in Figure 10) was observed when the temperature increased over ~120 °C for both cathodes. The two different decay rates of the QE indicate that the dissociation of the cathode material was primarily dependent on the substrate temperature. However, in the meantime, the reflectivity was initially decreased (when the temperature increased above ~130 °C) and then gradually increased (when the temperature rose above ~190 °C) for the thick cathode (KCsSb-5). In contrast, for the thin cathode (KCsSb-6), the reflectivity was continuously increased when the substrate temperature increased beyond 130 °C. This difference in the real-time reflectivity behavior during the cathode’s degradation process between the thick and thin cathodes was reproducible, and it indicates that these two kinds of photocathodes (i.e., thick and thin) may have possessed unique and varied crystal structures. By looking at the QE and reflectivity curves in Figure 10, it can be estimated that the cathode materials were almost entirely evaporated when the temperature increased over 230 °C and 420 °C for thin and thick cathodes, respectively. 

## 4. Density Functional Theory (DFT) Study

**Figure 11:**
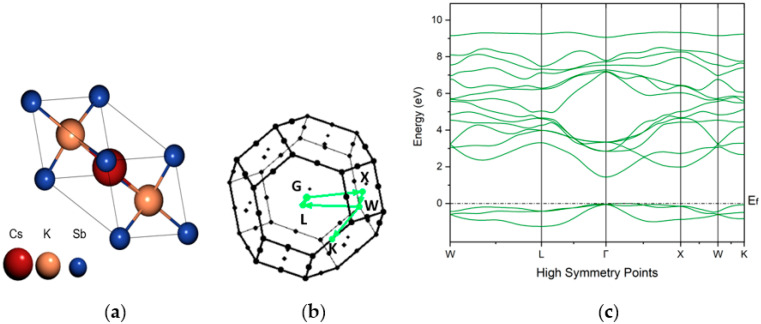
(**a**) Ball and stick representation of the unit cell of K_2_CsSb. (**b**) Brillouin zone of K_2_CsSb with high symmetry points and the path connecting them are highlighted in color. (**c**) Electronic band structure of K_2_CsSb by using the HSE functional. The Fermi energy ($E_{f}$) was set to zero at the valence band maximum.

To gain more insight into the electronic structure of this photo-emissive material, density functional theory (DFT) calculations were performed by using the QUANTUM ESPRESSO distribution [34,35]. These calculations were performed by assuming the cathodes had a monocrystalline structure. Although, photocathodes obtained experimentally may have different crystal structures and massive defects, modern density function theory, which is based on the first principles method, is capable of predicting the optical, electronic, and magnetic properties of materials with high accuracy. In this study, comparing the optical results obtained from the experimental method (such as reflectivity) and the results obtained from DFT simulation provided us with a better and more efficient strategy to understand and rationalize the properties of this kind of photoemissive material. Here, we focused on the face-cantered-cubic (FCC) phase (with a space group of F m-3-m) of the K_2_CsSb material and investigated the electronic and optical properties. By performing the volume optimization and fitting results with the Birch–Murnaghan equation [36,37], the lattice parameter a = 8.7587 Å was determined, which had good agreement with the previous experimental [38] and theoretical findings [39,40,41]. As shown in Figure 11a, the Sb atoms (depicted in blue color) were located at the origin of the cell at the Wyckoff position (0, 0, 0), while the alkali species Cs and K (depicted in red and orange colors, respectively) were located at the crystal coordinates (1/2, 1/2, 1/2) and ± (1/4, 1/4, 1/4), respectively. Here, in this work, the band structure calculation was performed by using both generalized gradient approximation (GGA) of the Perdew–Burke–Ernzerhof (PBE) functional [42] and hybrid Heyd–Scuseria–Ernzerh (HSE) functional, which incorporate the exact exchange [43]. To model the electron–ion interaction, optimized norm-conserving Vanderbilt (ONCV) pseudopotentials were used [44], and the Brillouin zone was sampled using a 4 × 4 × 4 k-mesh for the calculation. The plane wave kinetic energy cutoff of 70 Ry and a charge density plane wave cutoff of 280 Ry were used for this calculation. Maximally localized Wannier functions [45] were computed with the Wannier90 code and used to interpolate the band structure [46]. The band gap calculated using HSE functional was 1.49 eV (direct band gap at Γ); however, the PBE results unsurprisingly underestimated the gap and obtained a value of 0.84 eV (direct band gap at Γ). The band gap estimated through the HSE functional agrees with the previous DFT studies [47] and was very close to the experimental value, i.e., 1.2 eV [24].

**Figure 12:**
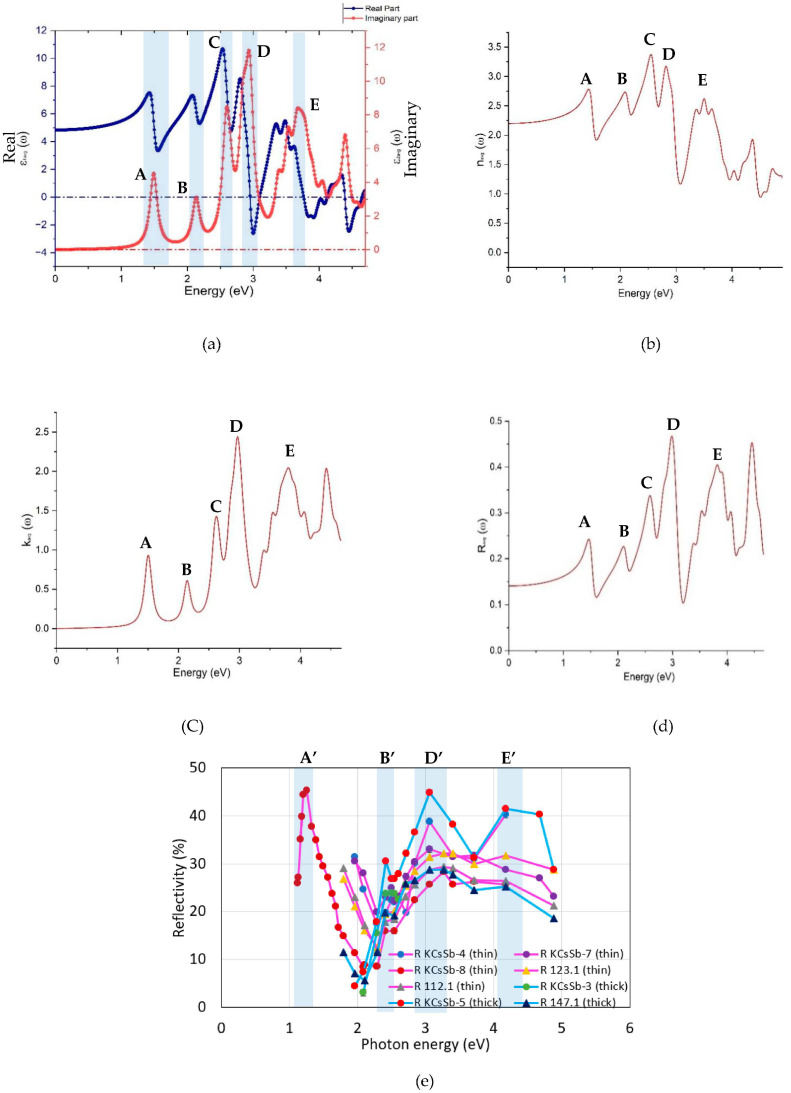
The optical spectra of the K_2_CsSb film as a function of the photon energy, where it represents the (**a**) real and imaginary parts of the dielectric function, (**b**) refractive index, (**c**) extinction coefficient, (**d**) reflectivity, and (**e**) experimental reflectivity of the produced K-Cs-Sb photocathodes (in the “R&D” and “production” system at LASA). Cathode 123.1 (thin), 112.1 (thin), and 147.1 (thick) were produced in the new “production” system [15]. Thick cathodes are highlighted by sky blue lines, whereas magenta lines in the plot highlight thin cathodes. Due to some technical problems, the full spectral reflectivity was only measured for the KCsSb-8 (thin) cathode.

The optical properties of the material were calculated using the epsilon.x code contained in the Quantum-Espresso package, where we determined the complex dielectric function $\epsilon \left(\omega \right)$ within the framework of the random phase approximation (RPA) [48] based on the DFT ground state calculation using the HSE functional. The dielectric function $\left(\epsilon \left(\omega \right)=\epsilon_{1}\left(\omega \right)+i\epsilon_{2}\left(\omega \right)\right)$ is a crucial parameter for exploring the optical and electronic properties of any semiconductor material. The imaginary part $\epsilon_{2}\left(\omega \right)$ is calculated from the momentum matrix elements between the occupied and unoccupied wave functions [49] and was computed by using the following Equation (2):

(2) $$\epsilon_{2}\left(\omega \right)=\frac{4\pi e^{2}}{{\Omega N_{k}m}^{2}}\sum_{n,k}\frac{\mathrm{df}(E_{k,n})}{dE_{k,n}}\frac{\eta \omega {\hat{M}}_{\alpha ,\beta }}{\omega^{4}+\eta^{2}\omega^{2}}+\ldots +\frac{8\pi e^{2}}{\Omega N_{k}m^{2}}\sum_{n\neq n^{’}}\sum_{k}\frac{{\hat{M}}_{\alpha ,\beta }}{E_{k,n^{’}}-E_{k,n}}....\frac{A\omega f(E_{k,n})}{\left[{\left(\omega_{k,n^{’}}-\omega_{k,n}\right)}^{2}-\omega^{2}\right]+A^{2}\omega^{2}}$$

where e denotes the electron charge; m represents the mass of the electron; Ω denotes the volume of the lattice cell; n and $n^{’}$ relate to the valance and conduction bands, respectively; $E_{k,n}$ are the eigenvalues of the Hamiltonian; $f(E_{k,n}$) is the Fermi distribution function that accounts for the occupation of the bands; ${\hat{M}}_{\alpha ,\beta }$ denotes the transition momentum from  α (valance with energy $E_{k,n}$) to β (conduction with energy $E_{k,n^{’}}$); A and η are coefficients; and $N_{k}$ is the number of electrons within the unit cell volume. With the help of the Kramers–Kronig relation (Equation (3)) and considering the imaginary part, the real part $\epsilon_{1}\left(\omega \right)$ of the complex dielectric function can be computed:

(3) $$\epsilon_{1}\left(\omega \right)=1+\frac{2}{\pi }P\int_{0}^{\infty }\frac{\omega^{\prime }\epsilon_{2}(\omega^{\prime })}{\omega^{\prime 2}-\omega^{2}}d\omega$$

where P represents the Cauchy principal value. By utilizing the real ($\epsilon_{1}\left(\omega \right)$) and imaginary ($\epsilon_{2}\left(\omega \right)$) parts of the dielectric function, we calculated the optical properties, such as the refractive index n(ω), extinction coefficient k(ω), and reflectivity R(ω) as a function of the incident photon energy using the following equation (Equations (4)–(6)), as presented in Figure 12b–d.

(4) $$n\left(\omega \right)=\left(\frac{1}{\sqrt{2}}\right){\left[\sqrt{{\varepsilon_{1}}^{2}\left(\omega \right)+{\epsilon_{2}}^{2}(\omega )}+\epsilon_{1}(\omega )\right]}^{\frac{1}{2}}$$

(5) $$k\left(\omega \right)=\left(\frac{1}{\sqrt{2}}\right){\left[\sqrt{{\varepsilon_{1}}^{2}\left(\omega \right)+{\epsilon_{2}}^{2}\left(\omega \right)}-\epsilon_{1}\left(\omega \right)\right]}^{\frac{1}{2}}$$

(6) $$R\left(\omega \right)=\frac{{\left(1-n\right)}^{2}+k^{2}}{{\left(1+n\right)}^{2}+k^{2}}$$

The imaginary part of the dielectric function $\epsilon_{2}\left(\omega \right)$ represents the absorption behavior that corresponds to the inter-band transitions (i.e., the transition from the valance band to the conduction band), whereas the real part of the dielectric function $\epsilon_{1}\left(\omega \right)$ represents the electronic polarization under incident light. The first peak, labeled A (in Figure 12a–d), appeared at around 1.49 eV, which corresponded to the direct optical (Γ–Γ) transition between the valance band maximum (VBM) and the conduction band minimum (CBM). At this point, the extinction coefficient k increased steeply, representing the fundamental absorption. The following peaks labeled B, C, D, and E lay in the visible–UV spectrum at approximately 2.13 eV, 2.60 eV, 2.93 eV, and 3.68 eV, respectively. Our results are in good agreement with the other DFT studies [50] and are closer to our experimental findings. Figure 12e shows the experimental reflectivity of K-Cs-Sb photocathodes produced in the R&D (thin: KCsSb-4, KCsSb-7, and KCsSb-8; thick: KCsSb-3 and KCsSb-5) and production system (thin: 123.1 and 112.1; thick: 147.1) at LASA. The first peak labeled A′ (in Figure 12e) appeared in the infrared spectrum at 1.22 eV, which may be construed as the fundamental absorption between the valance band maximum and conduction band minimum. This value is closer to our DFT reflectivity results (the first peak appeared at 1.49 eV due to the fundamental absorption between the valance band maximum and conduction band minimum) and coincided with the previous experimental findings (band gap of 1.2 eV) by Ghosh and Verma et al. [24]. The following peaks labeled B′, D′, and E′ appeared at approximately 2.40, 3.06–3.26 eV, and 4.17 eV, respectively, and have a reasonable agreement with our DFT results, where the maximum near to the 3 eV was particularly evident, as well as the reflectivity onset at about 1.22 eV, and near 2 and 4 eV, the peaks appeared. 

## 5. Conclusions

In this report, the details of the growth procedure of all produced photocathodes (in the R&D preparation system at INFN LASA, Milan, Italy), including the influence of temperature and thicknesses on the photocathode’s properties, are discussed. It was observed that the substrate temperature played a crucial role in the formation of the cathode film and affected its photoemissive and optical properties. Moreover, we observed that a different deposition rate could yield a better QE, especially for the K-Sb films. The reproducibility of the distinct behavior of reflectivity and color of the films indicated a potential variation in the chemical composition between the two kinds of photocathode material (i.e., thin and thick), which may have stemmed from different thicknesses of the Sb layer. However, an extensive surface characterization is required to further understand the detailed mechanism. The effect of laser heating and substrate temperature on the cathode’s degradation is also briefly discussed. In addition, the electronic and optical properties of the K_2_CsSb material were studied using DFT techniques. Our combined theoretical and experimental study shows that the correlation of the optical properties (such as reflectivity) helped us to understand and rationalize the photoemissive material’s properties. The photoemission threshold and band gap of these photocathodes were also determined. To obtain more insight into the evolution of the real-time photocurrent and reflectivity at different wavelengths during photocathode deposition, a “multi-wavelengths” diagnostic setup (i.e., the real-time photocurrent and reflectivity measurements at different wavelengths in the range from 254–690 nm) will be applied in the near future. This will help us to further understand the structural changes of the photocathodes during the deposition, and the results will be reported in forthcoming publications.

## Figures and Tables

**Table 1:** Summary of cathode growth parameters and evaporated thicknesses of Sb, K, and Cs. All the thicknesses were measured using a pre-calibrated quartz microbalance (QMB).

Cathode	Sb (nm)	K (nm)	Cs (nm)	K/Sb Ratio	Cs/Sb Ratio	TSb-TK-TCs (°C)	QE (%) (514 nm)	ΔR (%) Variation after K (543 nm)	ΔR (%) Variation after Cs (543 nm)
KCsSb-4	5 ± 0.9	41 ± 0.1	106 ± 0.5	8.2	21.2	90-90-90	3.9	47	37
KCsSb-6	5 ± 0.9	32 ± 0.1	117 ± 0.5	6.4	23.4	90-120-90	4.6	48.5	36.6
KCsSb-7	5 ± 0.9	34 ± 0.5	121 ± 0.5	6.8	24.2	90-120-110	5.4	48.33	38.7
KCsSb-8	5 ± 0.9	43 ± 0.5	31 ± 0.5 *	8.6	6.2 *	90-130-120	8.84	61.19	46.6 *
KCsSb-3	10 ± 0.9	66 ± 0.5	313 ± 0.9	6.6	31.3	60-60-60 **	5.2	65.4	0
KCsSb-5	10 ± 0.5	75 ± 0.1	316 ± 0.5	7.5	31.6	90-90-90	4.6	66.6	−7
NaKSb-2	9 ± 0.5	46 ± 0.1	-	5.1	-	90-120	0.3 #	68.8	-

* Due to some technical problem, the Cs deposition was not fully completed for the KCsSb-8 cathode. ** The substrate temperature was increased from 60 °C to 90 °C during the Cs evaporation for the KCsSb-3 cathode, as reported in [20]; # QE is shown only for the K-Sb phase.

**Table 2:** Summary of the absolute % QE increase after turning off the Cs source and corresponding substrate temperature. * The substrate temperature was increased from 60 °C to 90 °C during the Cs evaporation for the KCsSb-3 cathode, as reported in [20].

Cathode	Temperature (°C) during Cs Deposition	Absolute % QE Increase (after Turning off the Cs Source)
KCsSb-3	60 *	308
KCsSb-4	90	57
KCsSb-5	90	55
KCsSb-6	90	63
KCsSb-7	110	30
